# Risk factors of recurrence after robot-assisted laparoscopic partial nephrectomy for solitary localized renal cell carcinoma

**DOI:** 10.1038/s41598-023-51070-8

**Published:** 2024-02-23

**Authors:** Jae Hoon Chung, Wan Song, Minyong Kang, Hyun Hwan Sung, Hwang Gyun Jeon, Byong Chang Jeong, Seong Soo Jeon, Hyun Moo Lee, Seong IL Seo

**Affiliations:** Department of Urology, Samsung Medical Center, Sungkyunkwan University School of Medicine, Seoul, Republic of Korea

**Keywords:** Renal cancer, Renal cell carcinoma

## Abstract

To evaluate the recurrence rate and risk factors of recurrence after robot-assisted laparoscopic partial nephrectomy for solitary renal cell carcinoma (RCC). A total of 1265 cases of initial solitary localized RCC were analyzed. The baseline characteristics, complexity (REANL nephrometry score), intra- and peri-operative outcomes, and recurrence were evaluated. Logistic regression was performed to evaluate the factors affecting recurrence after RAPN for solitary localized RCC. Recurrence after robot-assisted partial nephrectomy (RAPN) occurred in 29 patients (2.29%). The median follow-up was 36.0 months. The N domain (nearness to collecting system/sinus) (odd ratio (OR) 3.517, 95% confidence interval (CI) 1.557–7.945, p = 0.002), operation time (OR 1.005, 95% CI 1.001–1.010, p = 0.013), and perioperative transfusion (OR 5.450, 95% CI 1.197–24.816, p = 0.028) affected recurrence. Distant metastasis among patients with recurrence was significantly associated with nearness to the collecting system/sinus (OR 2.982, 95% CI 1.162–7.656, p = 0.023) and distance between the mass and collecting system/sinus (OR 0.758, 95% CI 0.594–0.967, p = 0.026). Nearness to the collecting system/sinus, operation time, and perioperative transfusion affect recurrence after RAPN for solitary localized RCC. Moreover, the proximity to the collecting system/sinus and distance between the mass and collecting system/sinus were significantly related to distant metastasis after RAPN.

## Introduction

With the recent increase in health evaluations using ultrasound or computed tomography (CT), renal cell carcinoma (RCC) is mostly diagnosed at an early stage^1^. Partial nephrectomy (PN) has become the standard treatment for localized RCC over radical nephrectomy due to the equivalent oncological outcomes and advantages of nephron sparing^2^. The decision whether to perform radical nephrectomy or PN as a treatment for localized RCC is determined by the experience of the operator, T stage, and renal mass complexity^3^. In the past, as technical hurdles existed for PN, resulting in a high incidence of adverse sequelae such as chronic renal disease, radical nephrectomy was overused^4,5^. However, due to recent advances in surgeon proficiency and robotic surgery, PN is performed not only for small RCCs less than 2 cm but also for large RCCs of T1b/T2^6^. Even in the case of large RCCs, PN showed similar oncological outcomes and better functional preservation than radical nephrectomy; therefore, PN should be recommended^7^. Moreover, robot-assisted partial nephrectomy (RAPN) has now become the first choice of treatment for RCC below T2 due to the popularization of robotic surgery.

In RCC, there are only some reports on local recurrence or distant metastasis rates after PN, with recurrence rates varying from 1 to 40%^8^. Moreover, the focus has been on the feasibility and oncological outcomes of PN compared with radical nephrectomy^9,10^. However, the recurrence rate and risk factors after RAPN as a treatment for solitary RCC have not been reported. Recently, a systematic review by Henderickx et al. reported that a positive surgical margin (PSM) in pT1 RCC could increase the risk of recurrence after partial nephrectomy^11^. However, the risk of bias of the analyzed previous studies was high, and the high heterogeneity of this study made it difficult to evaluate as an optimistic systematic review.

In the present study, we assessed the recurrence rate and risk factors after RAPN as a treatment for solitary RCC through a single-surgeon large-scale observational study.

## Results

### Demographics

**Figure 1:**
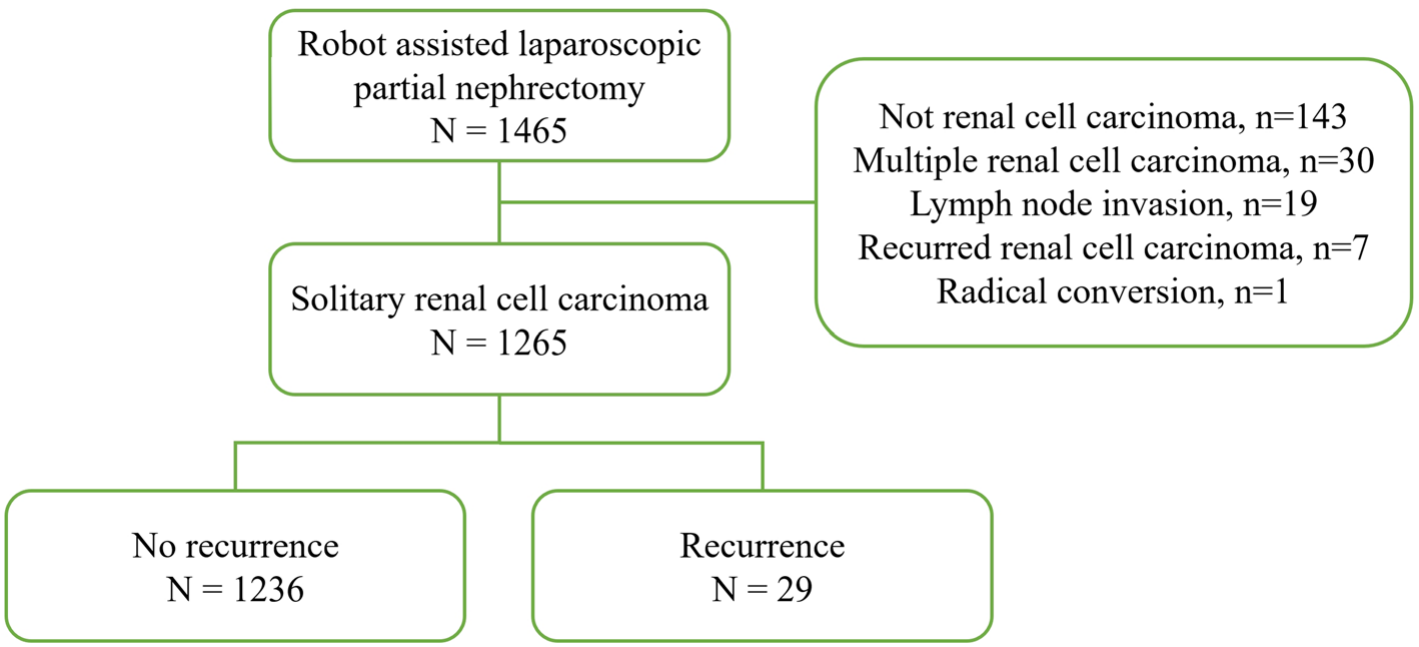
Flow sheet of inclusion.

Among the 1465 patients who underwent RAPN, 1265 met the inclusion/exclusion criteria, and recurrence occurred in 29 (2.29%) of them (Fig. 1). The median follow-up period of the 1265 patients was 36.0 months. The mean ages of patients in the no recurrence and recurrence groups were 52.87 ± 12.06 and 52.10 ± 12.32, respectively (p = 0.742). Hypertension was present in 33.25% (411/1236) and 51.72% (15/29) of the no recurrence and recurrence group, respectively (p = 0.037). A Family history of RCC was present in 3.24% (24/1236) of the no recurrence group, and no patients in the recurrence group had a familial history (p = 0.005). There was no significant difference in renal function between the two groups. Regarding clinical stage, 34.48% (10/29) of the recurrence group were T1b, 13.51% (167/1236) were T1b, and 0.49% (6/1236) were T2a in the no recurrence group (p = 0.005). According to the nephrometry score, 45.95% (568/1236) were at intermediate risk, 8.50% (105/1236) were at high risk in the recurrence group, 55.17% (16/29) were at intermediate risk, and 24.14% (7/29) were at high risk in the no-recurrence group (p = 0.003) (Table 1).

**Table 1:** Baseline characteristics.

Parameters	No recurrence (n = 1236)	Recurrence (n = 29)	p-value
Age, yrs	52.87 ± 12.06	52.10 ± 12.32	0.742
Sex, male	821 (66.42%)	22 (75.86%)	0.287
BMI, kg/m^2^	25.54 ± 7.30	26.54 ± 3.90	0.196
ASA classification, n			0.686^†^
1	370 (29.94%)	6 (20.69%)	
2	804 (65.05%)	22 (75.86%)	
3	60 (4.85%)	1 (3.45%)	
4	2 (0.16%)	0	
Smoking, pack years	6.56 ± 18.05	5.14 ± 8.82	0.620
Hypertension, n	411 (33.25%)	15 (51.72%)	0.037^†^
Diabetes mellitus, n	156 (12.62%)	6 (20.69%)	0.199^†^
Familial history, n	24 (3.24%)	0	0.005^†^
Renal function test, DTPA			
Right, ml/min	42.06 ± 11.71	41.54 ± 14.62	0.854
Left, ml/min	43.72 ± 11.78	42.14 ± 14.68	0.583
Normalized GFR, ml/min	82.81 ± 21.67	77.01 ± 24.34	0.223
Creatinine, mg/dl	0.85 ± 0.21	0.92 ± 0.21	0.079
Estimated GFR, ml/min/1.73 m^2^	91.49 ± 16.60	84.44 ± 18.99	0.057
Clinical T stage			0.005^†^
T1a	1063 (86.00%)	19 (65.52%)	
T1b	167 (13.51%)	10 (34.48%)	
T2a	6 (0.49%)	0	
Nephrometry score			0.003^†^
Low risk	550 (44.50%)	6 (20.69%)	
Intermediate risk	568 (45.95%)	16 (55.17%)	
High risk	105 (8.50%)	7 (24.14%)	
Laterality, right	627 (50.73%)	11 (37.93%)	0.173^†^
Follow up, months	39.64 ± 32.03	40.38 ± 33.10	0.906

*ASA* American Society of Anesthesiologists, *DTPA* Diethylenetriamine pentaacetate renal scan, *GFR* Glomerular filtration rate.

Student’s t test, ^†^Chi-square test.

The operation times were 261.14 ± 61.66 and 195.15 ± 87.03 min in the recurrence and no recurrence groups, respectively (p < 0.001). Perioperative transfusion was performed in 10.34% (3/29) and 1.46% (18/1236) of the recurrence and no recurrence groups, respectively (p < 0.001). As for the pathologic stage, 20.69% (6/29) were T1b and 6.90% (2/29) were T3a in the recurrence group. In the no recurrence group, 15.05% (186/1236) were T1b, 7.28% (9/1236) were T2a, and 1.46% (18/1236) were T3a (p = 0.005). In terms of Fuhrmann grade, 60.03% (742/1236) of the patients in the no recurrence group and 41.38% (12/29) of the patients in the recurrence group were low-grade (p = 0.035) (Table 2).

**Table 2:** Surgical and oncological outcomes.

	No recurrence (n = 1236)	Recurrence (n = 29)	p-value
Surgical outcomes			
Retroperitoneal approach	429 (34.71%)	10 (34.48%)	0.934
Operation times, mins	195.15 ± 87.03	261.14 ± 61.66	< 0.001
Ischemic time, mins	20.33 ± 7.96	23.66 ± 8.78	0.052
Estimated blood loss, ml	139.41 ± 118.55	175.36 ± 179.00	0.300
Capsular incision	37 (2.99%)	2 (6.90%)	0.229
Transfusion, n	18 (1.46%)	3 (10.34%)	< 0.001
Hospital stay, days	5.92 ± 2.67	6.86 ± 2.05	0.021
Peri-operative complications, n	5 (0.40%)	0	0.731
Oncological outcomes			
Histology			0.811
Clear cell carcinoma	1021 (82.61%)	27 (93.10%)	
Papillary	91 (7.36%)	0	
Chromophobe	109 (8.82%)	2 (6.90%)	
Others	15 (1.21%)	0	
Pathologic T stage			0.005
T1a	1023 (82.77%)	21 (72.41%)	
T1b	186 (15.05%)	6 (20.69%)	
T2a	9 (7.28%)	0	
T3a	18 (1.46%)	2 (6.90%)	
Fuhrmann grade			0.035
I	48 (3.88%)	1 (3.45%)	
II	694 (56.15%)	11 (37.93%)	
III	477 (38.59%)	17 (58.62%)	
IV	13 (1.05%)	0	
Tumor size, cm	2.80 ± 1.33	3.38 ± 1.33	0.027
Safety margin, mm	4.11 ± 3.82	4.29 ± 3.25	0.779
Margin involvement	6 (0.49%)	0	0.712

Student’s t test, Chi-square test.

### Recurrence after RAPN

**Figure 2:**
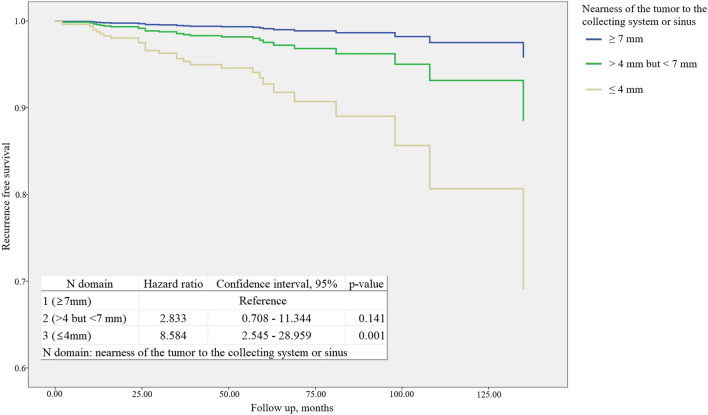
Cox regression for recurrence free survival according to nearness of the tumor to the collecting system or sinus. Mean recurrence free survival: 155.04 ± 1.22(≥ 7 mm), 135.74 ± 1.98 (> 4 mm but < 7 mm) and 129.90 ± 4.14 (≤ 4 mm) (p < 0.001).

On multivariate analysis, N domain (nearness to collecting system/sinus) (OR 3.517, 95% CI 1.557–7.945, p = 0.002), operation time (OR 1.005, 95% CI 1.001–1.010, p = 0.013), and perioperative transfusion (OR 5.450, 95% CI 1.197–24.816, p = 0.028) were significantly related to recurrence (Table 3). According to N domain classification, the mean recurrence free survival period was 155.04 ± 1.22 months (≥ 7 mm), 135.74 ± 1.98 months (> 4 mm but < 7 mm), and 129.90 ± 4.14 months (≤ 4 mm) (p < 0.001). The hazard ratio of recurrence is 2.833 (95% CI 0.708–11.344, p = 0.141) in > 4 mm but < 7 mm group and 8.584 (95% CI 2.545–28.959, p = 0.001) (Fig. 2).

**Table 3:** Logistic regression analysis for tumor recurrence after robot assisted laparoscopic partial nephrectomy for solitary renal cell carcinoma.

Variables	Univariate analysis			Multivariate analysis		
	OR	CI, 95%	p-value	OR	CI, 95%	p-value
Age	0.995	0.965–1.025	0.723			
Sex	1.589	0.673–3.749	0.291			
Body mass index	1.009	0.984–1.035	0.499			
Smoking, pack-year	0.996	0.962–1.030	0.799			
ASA classification	1.295	0.654–2.567	0.459			
Hypertension	2.151	1.028–4.498	0.042	1.674	0.755–3.715	0.205
Diabetes mellitus	1.806	0.724–4.505	0.205			
Serum Creatinine	1.406	0.763–2.593	0.275			
Estimated GFR	0.976	0.957–0.997	0.022	0.991	0.968–1.014	0.435
Laterality	1.685	0.789–3.596	0.178			
Clinical T stage	3.350	1.531–7.330	0.002	1.504	0.503–4.495	0.465
Nephrometry						
Low risk						
Intermediate risk	2.582	1.003–6.647	0.049	1.753	0.294–10.448	0.537
High risk	6.111	2.014–18.547	0.001	0.671	0.242–1.857	0.442
Radius	1.634	0.815–3.276	0.167			
Exophytic/endophytic	1.290	0.792–2.102	0.306			
Nearness to collecting system/sinus	2.978	1.728–5.135	< 0.001	3.517	1.557–7.945	0.002
Anterior	Reference					
Posterior	1.099	0.265–4.557	0.897			
x	0.557	0.099–3.136	0.507			
Location relative to the polar lines	1.539	1.135–2.089	0.006	1.223	0.832–1.796	0.306
Approach, retroperitoneal	0.968	0.446–2.100	0.934			
Ischemic time	1.044	1.005–1.085	0.025	0.972	0.917–1.032	0.354
Capsular incision	2.400	0.550–10.472	0.244			
Operation time	1.007	1.003–1.010	< 0.001	1.005	1.001–1.010	0.013
Estimated blood loss	1.002	1.000–1.004	0.116			
Peri-operative transfusion	7.808	2.165–28.152	0.002	5.450	1.197–24.816	0.028
Safety margin	1.006	0.915–1.107	0.899			
Tumor size	1.305	1.039–1.639	0.022	0.877	0.571–1.348	0.550
Histology	0.644	0.297–1.393	0.264			
Fuhrman grade	1.558	0.826–2.940	0.171			
High grade	2.414	1.136–5.130	0.022	1.943	0.887–4.254	0.097
Capsule invasion	1.771	0.605–5.188	0.297			
Fat invasion	5.469	0.661–45.213	0.115			
Sinus invasion	8.764	0.991–77.490	0.051			
Pathologic T stage	1.497	1.035–2.167	0.032	1.255	0.747–2.111	0.391

*ASA* American Society of Anesthesiologists, *CI* confidence interval, *GFR* Glomerular filtration rate, *OR* odds ratio.

**Figure 3:**
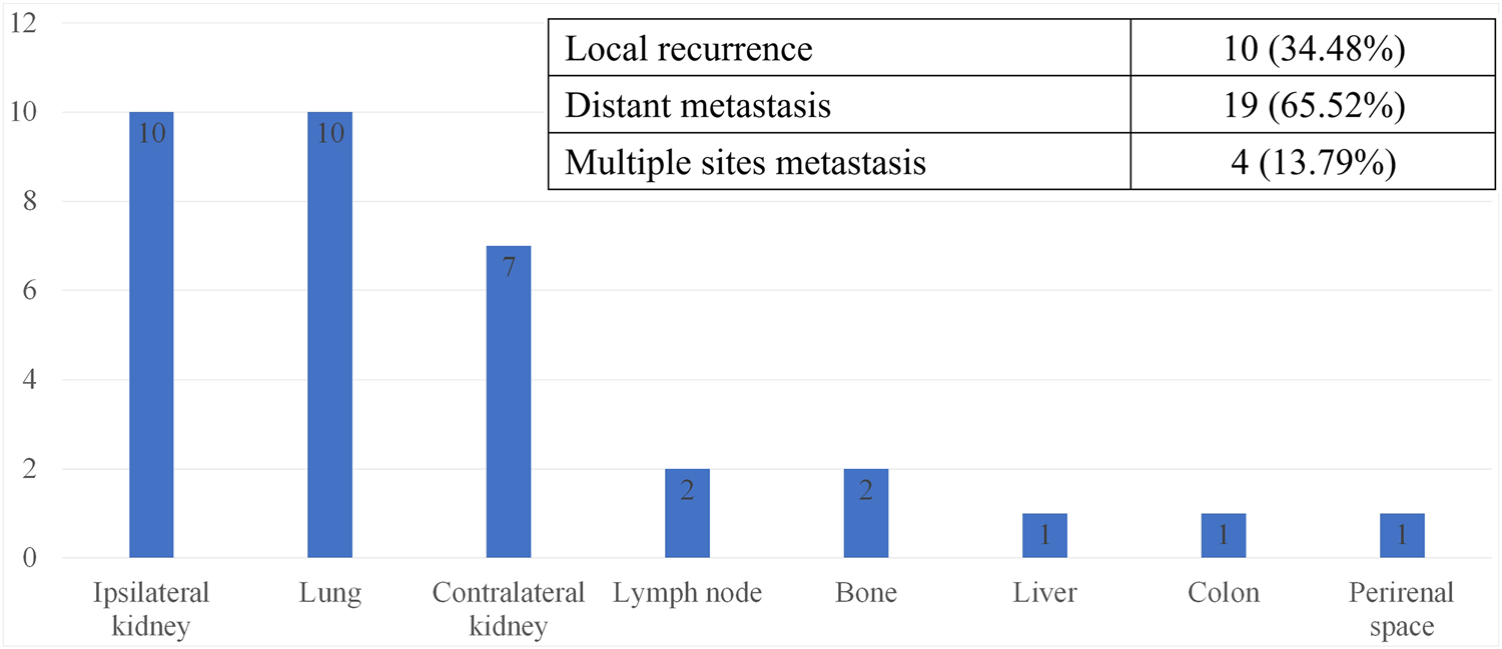
Site of recurrence lesions.

Among the 29 patients with recurrence, 34.48% (10/29) had local recurrence in the ipsilateral kidney and 65.52% (19/29) had distant metastasis. In addition, metastasis to multiple sites was observed in 13.79% (4/29) of the patients (Fig. 3). Distant metastasis among patients with recurrence was significantly associated with nearness to the collecting system/sinus (OR 2.982, 95% CI 1.162–7.656, p = 0.023) and distance between the mass and the collecting system/sinus (OR 0.758, 95% CI 0.594–0.967, p = 0.026) (Table 4) (Supplementary 3).

**Table 4:** Logistic regression analysis for distant metastasis in tumor recurrence after robot assisted laparoscopic partial nephrectomy for solitary renal cell carcinoma (n = 29).

Variables	Univariate analysis		
	OR	CI 95%	p-value
N domain	2.982	1.162–7.656	0.023
Length between mass and collecting system/sinus	0.758	0.594–0.967	0.026

*OR* odds ratio, *CI* confidence interval.

## Discussion

Nearness to the collecting system/sinus, operation time, and perioperative transfusion affect recurrence after RAPN. In particular, proximity to the collecting system/sinus and the distance between the mass and collecting system/sinus are significantly related to distant metastasis.

As a treatment for clinically localized small renal masses, PN showed similar surgery-related mortality, cancer-specific survival, and time-to-recurrence with superiority in decreased time-to-death from any cause compared to radical nephrectomy^12^. Moreover, compared to radical nephrectomy, PN as a treatment for large renal masses (T1b or T2) also has equivalent cancer control and better preservation of renal function with the potential for better long-term survival^13^.

However, there are few studies on the risk factors for recurrence after PN for RCC. Recently, through a systematic review, Henderickx et al. reported that a PSM was a risk factor for local recurrence; the percentage of cases with a PSM ranged from 0 to 34.4% and local recurrence varied from 0 to 9.1%^11^. In the present study, PSM was defined as the presence of malignant cells at the surgical margin in the pathologic report, and only six RAPN patients (0.41%) were evaluated as having a PSM. All six patients had no recurrence during follow-up. Khalifey et al. reported that no factor, including tumor size, pathological stage, tumor grade, multiple tumors, or surgeon learning curve, could predict a PSM after partial nephrectomy^14^. However, in RAPN, the surgeon factor cannot be avoided in PSM. Although the association between recurrence and PSM could not be clearly confirmed due to the very low incidence of PSMs in this study, reducing PSMs is needed for better oncological outcomes.

Tumor incision is a known risk factor for tumor recurrence and metastasis^15^. However, in our results, there was no significant relationship between capsular incision and tumor recurrence. Moreover, a retrospective study by Ito et al. reported that capsular incision during PN was not associated with poor oncological outcomes^16^. However, in both results, the number of capsular incisions was too small to draw definitive conclusions. Yoshino et al. reported that RCC cells remained on the surface of the scissors after capsular incision, and elimination of the tumor cell using monopolar electrical treatment is needed^17^. In addition, Li et al. suggested that capsular incision would affect tumor recurrence and recommended that scissors be treated with povidone-iodine after capsular incision^18^. Although there is no definite conclusion regarding the effect of capsular incision on recurrence, if capsular incision occurs, it is necessary to secure a safe surgical margin through additional resection and treatment of the scissor surface. In this study, the incidence of capsular incision and PSM was low, and appropriate evaluations could not be assessed. However, avoiding obvious risk factors such as tumor violation or margin status could improve oncological outcomes.

In our results, the N domain (nearness to collecting system/sinus) in the RENAL nephrometry score was significantly related to recurrence. Maxwell et al. also reported that the N domain had a significant effect on recurrence after thermal ablation (hazard ratio 3.15, 95% confidence intervals 1.31–7.62, p < 0.0001)^19^. Unlike the present study, they reported that the R domain (the diameter of the mass) was associated with recurrence. This may be due to the difference in the treatment characteristics of ablation and surgical resection. Additionally, propensity score matching was performed for familial history, hypertension, and clinical T stage to further assess the risk of N domain recurrence. After matching, there was no statistical difference in the baseline characteristics (Supplementary 1), and through logistic regression, the N domain was the only factor that significantly affected recurrence (Supplementary 2).

In the present study, operation time also affected recurrence. This may be due to the longer tumor manipulation time than the surgical time itself. Wan et al. reported that high plasma cell-free DNA levels were associated with a significantly higher recurrence rate in clear cell RCC^20^. Although it has not been reported that tumor manipulation increases circulating tumor cells during PN, it is known that surgical management causes dissemination of circulating tumor cells^21^. Even in the case of RCC, if the tumor manipulation time is prolonged, the level of circulating tumor cells may increase, which could affect recurrence. Moreover, nearness to the collecting system/sinus and the distance between the mass and collecting system/sinus were associated with distant metastasis in our results. It is possible that the level of circulating tumor cells during surgery might increase more when the sinus and tumor are closer. Further evaluation using a prospective study is required.

Abu-Ghanem et al. reported that perioperative blood transfusion was associated with reduced recurrence-free, cancer-specific, and overall survival in patients undergoing nephrectomy for RCC^22^. Our results also showed that perioperative blood transfusion was associated with tumor recurrence after RAPN. The mechanism of the adverse oncological effects in transfusion may be related to the suppressive effects on the immune system^23,24^. Moreover, transient immune impairment, which comes from transfusion, may enhance a condition favorable to cancer cells^25^. However, there is still controversy regarding transfusion and oncological outcomes in RCC.

This was a retrospective study, and its limitation was the relatively short follow-up period. Moreover, whether the recurrence in ipsilateral RCC was an incidental lesion or a metastatic lesion was not clearly determined. However, a pathological review was performed in all 10 cases, and the histology was confirmed to be the same as that of previous RCC on biopsy. The bias may have been reduced with the data of a single expert surgeon, which can reduce the surgeon factor. In addition, this study is significant in that it is the first large-scale study to evaluate risk factors for recurrence after RAPN as a treatment for solitary RCC.

## Methods

### Patients

A total of 1465 patients who underwent RAPN between 2008 and 2022 were retrospectively analyzed. RAPN was performed by a single expert surgeon. RAPN was performed when it was determined that nephron-sparing surgery was possible for a localized renal mass of T1 or T2 stage. We excluded patients with multiple renal masses, non-RCC, lymph node invasion, and recurrent masses from this study.

Among these patients, 1265 cases of initial solitary localized RCC were selected based on pathological reports (Fig. 1). To evaluate the recurrence risk factors after RAPN as a treatment for solitary RCC, the recurrence and no recurrence groups were compared.

### Clinicopathologic assessment

The baseline characteristics of the patients, including age at RAPN, underlying disease, familial history, routine laboratory test, and diethylenetriamine pentaacetic acid renal scan were evaluated. Abdominal computed tomography (CT) with/without magnetic resonance imaging (MRI) was performed on the patient, and complexity was evaluated using the RENAL nephrometry score^26^. Estimated blood loss, warm ischemic time, capsular incision, and intraoperative complications, including transfusion, were evaluated. In addition, the length of hospital stay and perioperative complications were evaluated. The histopathology was evaluated by experienced uropathologists.

### Follow up

After RAPN, the patients were generally followed up every 3–6 months in the first year. Thereafter, follow-up was performed at intervals of 6–12 months. Patients underwent abdominal CT or MRI, chest radiography, and routine laboratory tests at each visit. Recurrence-free survival was defined as the interval between the date of surgery and the time of the first tumor recurrence. The cause of death was determined by the physicians responsible and death certificates.

### Surgical approach

The operation was performed using a four-arm da Vinci Robotic System (Intuitive Surgical, Seoul, Korea), using five ports. Based on the location of the renal mass, a retroperitoneal or transperitoneal approach was performed according to the surgeon's decision. The operation was performed through main artery clamping without selective ischemia, and PN was performed under warm ischemia in all patients. In most cases, tumor excision uses a modified tumor enucleation method with a safety margin of less than 1 cm through blunt dissection and incision using monopolar scissors. A capsular incision was defined as a case where tumor violation occurred due to an unintentional incision in the mass during tumor excision. When a capsular incision was made, the safety margin was secured by additional resection. For the tumor bed, continuous running suture was performed through barbed suture, and renorrhaphy was also performed using barbed suture. Warm ischemia was maintained from mass excision to the completion of renorrhaphy.

### Statistical analysis

The baseline characteristics were compared between the patients with and without recurrence after RAPN using the chi-square test for categorical variables and the independent t-test for continuous variables. Kaplan–Meier survival analysis was used to calculate the estimates for recurrence-free survival. Logistic regression analysis was performed to evaluate the factors affecting recurrence after RAPN for solitary localized RCC.

### Ethics statement

This study was conducted in accordance with the Declaration of Helsinki and current ethical guidelines. The Institutional Review Board of Samsung Medical Center approved the current study (approval number: 2023-03-053). All methods were conducted in accordance with relevant guidelines and regulations. The requirement for written informed patient consent was waived by the Institutional Review Board of the Samsung Medical Center due to the retrospective nature of the study. Personal identifiers were completely deleted to ensure that the data were analyzed anonymously.

## Conclusions

Nearness to the collecting system/sinus, operation time, and perioperative transfusion affect recurrence after RAPN for solitary localized RCC. Moreover, the proximity to the collecting system/sinus and distance between the mass and collecting system/sinus were significantly related to distant metastasis after RAPN.

### Supplementary Information

Supplementary Information.

## Data Availability

The raw data for this study are available upon reasonable request from the corresponding author.
