# Supplementary material for: Risk factors of recurrence after robot-assisted laparoscopic partial nephrectomy for solitary localized renal cell carcinoma
Source: Sci Rep. 2024 Feb 23;14:4481. doi: 10.1038/s41598-023-51070-8 (PMC10891047; doi:10.1038/s41598-023-51070-8)
Supplement: Supplementary file 1 — Supplementary Information. [file 41598_2023_51070_MOESM1_ESM.docx]

| Supplementary 1. Baseline characteristics after propensity score matching | | | |
| --- | --- | --- | --- |
| Parameters | No recurrence (n=29) | Recurrence (n=29) | p-value |
| Age, yrs. | 55.97±13.51 | 52.10±12.32 | 0.260 |
| Sex, male | 18 (62.07%) | 22 (75.86%) | 0.395^†^ |
| BMI, kg/m^2^ | 25.96±2.78 | 26.54±3.90 | 0.522 |
| ASA classification, n |  |  | 1.000^†^ |
| 1 | 6 (20.69%) | 6 (20.69%) |  |
| 2 | 22 (75.86%) | 22 (75.86%) |  |
| 3 | 1 (3.45%) | 1 (3.45%) |  |
| Smoking, pack years | 2.98±6.52 | 5.14±8.82 | 0.356 |
| Hypertension, n | 15 (51.72%) | 15 (51.72%) | 1.000^†^ |
| Diabetes mellitus, n | 4 (13.79%) | 6 (20.69%) | 0.730^†^ |
| Renal function test, DTPA |  |  |  |
| Right, ml/min | 43.86±13.50 | 41.54±14.62 | 0.547 |
| Left, ml/min | 44.58±11.07 | 42.14±14.68 | 0.496 |
| Normalized GFR, ml/min | 86.25±23.52 | 77.01±24.34 | 0.163 |
| Creatinine, mg/dl | 0.83±0.21 | 0.92±0.21 | 0.098 |
| Estimated GFR, ml/min/1.73m^2^ | 93.00±20.59 | 84.44±18.99 | 0.106 |
| Clinical T stage |  |  | 1.000^†^ |
| T1a | 20 (68.97%) | 19 (65.52%) |  |
| T1b | 9 (31.03%) | 10 (34.48%) |  |
| Nephrometry score |  |  | 0.143^†^ |
| Low risk | 12 (41.38%) | 6 (20.69%) |  |
| Intermediate risk | 13 (44.83%) | 16 (55.17%) |  |
| High risk | 3 (10.34%) | 7 (24.14%) |  |
| Laterality, right | 17 (58.62%) | 11 (37.93%) | 0.189^†^ |
| Follow up, months | 58.76±38.08 | 40.38±33.10 | 0.055 |
| ASA: American Society of Anesthesiologists, DTPA: Diethylenetriamine pentaacetate renal scan, GFR: Glomerular filtration rate Mann Whiteny U test, ^†^Fisher’s exact test | | | |

| Supplementary 2. Logistic regression analysis for tumor recurrence after robot assisted laparoscopic partial nephrectomy for solitary renal cell carcinoma, propensity score matching group | | | | |
| --- | --- | --- | --- | --- |
| Variables | | Univariate analysis | | |
|  |  | OR | CI, 95% | p-value |
| Age |  | 0.976 | 0.937-1.018 | 0.257 |
| Sex |  | 0.521 | 0.168-1.618 | 0.259 |
| Body mass index | | 1.053 | 0.900-1.232 | 0.516 |
| Smoking, pack-year | | 1.039 | 0.961-1.123 | 0.337 |
| ASA classification | | 1.000 | 0.327-3.061 | 1.000 |
| Hypertension |  | 1.000 | 0.357-2.801 | 1.000 |
| Diabetes mellitus | | 1.630 | 0.408-6.521 | 0.489 |
| Serum Creatinine | | 8.815 | 0.647-120.025 | 0.102 |
| Estimated GFR | | 0.978 | 0.951-1.005 | 0.110 |
| Laterality |  | 2.318 | 0.809-6.644 | 0.118 |
| Clinical T stage | | 1.170 | 0.390-3.506 | 0.780 |
| Nephrometry | Low risk |  |  |  |
|  | Intermediate risk | 2.462 | 0.724-8.364 | 0.149 |
|  | High risk | 4.667 | 0.878-24.796 | 0.071 |
| Radius |  | 1.000 | 0.339-2.953 | 1.000 |
| Exophytic/endophytic | | 1.503 | 0.763-2.960 | 0.239 |
| Nearness to collecting system/sinus | | 3.297 | 1.589-6.840 | <0.001 |
| Anterior | | Reference | | |
| Posterior | | 1.833 | 0.522-6.445 | 0.345 |
| x | | 0.556 | 0.146-2.114 | 0.389 |
| Location relative to the polar lines | | 1.649 | 0.900-3.021 | 0.106 |
| Approach, retroperitoneal | | 1.382 | 0.452-4.225 | 0.571 |
| Ischemic time |  | 1.054 | 0.972-1.144 | 0.203 |
| Capsular incision | | 1.74E+09 | N/A | 0.999 |
| Operation time | | 1.004 | 0.998-1.011 | 0.198 |
| Estimated blood loss | | 1.002 | 0.998-1.006 | 0.418 |
| Peri-operative transfusion | | 1.80E+09 | N/A | 0.999 |
| Safety margin |  | 0.919 | 0.809-1.044 | 0.193 |
| Tumor size |  | 1.167 | 0.775-1.757 | 0.461 |
| Histology |  | 0.567 | 0.228-1.412 | 0.223 |
| Fuhrman grade | | 1.093 | 0.478-2.502 | 0.833 |
| High grade |  | 2.014 | 0.706-5.744 | 0.190 |
| Capsule invasion | | 0.768 | 0.184-3.206 | 0.717 |
| Fat invasion |  | 1.000 | 0.060-16.0791 | 1.000 |
| Sinus invasion | | 1.67E+09 | N/A | 1.000 |
| Pathologic T stage | | 1.128 | 0.643-1.981 | 0.674 |
|  | | | | |

| Supplementary 3. List of the length between tumor and collecting system/sinus in recurrence group | |
| --- | --- |
| Number of patients | Length between tumor and collecting system/sinus, mm |
| # 1-14 | 0 |
| # 15 | 1 |
| # 16, 17 | 2 |
| # 18, 19 | 4 |
| # 20, 21 | 5 |
| # 22 - 25 | 7 |
| # 26 | 8 |
| # 27, 28 | 10 |
| # 29 | 19 |
